# Population‐specific transcriptional differences associated with freeze tolerance in a terrestrial worm

**DOI:** 10.1002/ece3.3602

**Published:** 2018-03-11

**Authors:** Tjalf E. de Boer, Dick Roelofs, Riet Vooijs, Martin Holmstrup, Mónica J. B. Amorim

**Affiliations:** ^1^ Microlife Solutions Amsterdam The Netherlands; ^2^ Department of Ecological Science Faculty of Earth and Life Sciences VU University, Amsterdam Amsterdam The Netherlands; ^3^ Department of Bioscience Aarhus University Silkeborg Denmark; ^4^ Department of Biology and CESAM (Centre for Environmental and Marine Studies) University of Aveiro Aveiro Portugal

**Keywords:** cryoprotectant, membrane lipid, oxidative stress, RNAseq, sodium transport, transcriptional plasticity

## Abstract

*Enchytraeus albidus* is a terrestrial earthworm widespread along the coasts of northern Europe and the Arctic. This species tolerates freezing of body fluids and survives winters in a frozen state. Their acclimatory physiological mechanisms behind freeze tolerance involve increased fluidity of membrane lipids during cold exposure and accumulation of cryoprotectants (glucose) during the freezing process. Gene regulatory processes of these physiological responses have not been studied, partly because no gene expression tools were developed. The main aim of this study was to understand whether the freeze tolerance mechanisms have a transcriptomic basis in *E. albidus*. For that purpose, first the transcriptome of *E. albidus* was assembled with RNAseq data. Second, two strains from contrasting thermal environments (Germany and Greenland) were compared by mapping barcoded RNAseq data onto the assembled transcriptome. Both of these strains are freeze tolerant, but Greenland is extremely freeze tolerant. Results showed more plastic responses in the Greenland strain as well as higher constitutive expression of particular stress response genes. These altered transcriptional networks are associated with an adapted homeostasis coping with prolonged freezing conditions in Greenland animals. Previously identified physiological alterations in freeze‐tolerant strains of *E. albidus* are underpinned at the transcriptome level. These processes involve anion transport in the hemolymph, fatty acid metabolism, metabolism, and transport of cryoprotective sugars as well as protection against oxidative stress. Pathway analysis supported most of these processes, and identified additional differentially expressed pathways such as peroxisome and Toll‐like receptor signaling. We propose that the freeze‐tolerant phenotype is the consequence of genetic adaptation to cold stress and may have driven evolutionary divergence of the two strains.

## 1 INTRODUCTION

The majority of Enchytraeids are soil and litter dwelling oligochaetes widespread in Arctic and temperate regions. In particular, in the colder regions, the soil layers in which enchytraeids are living freeze every winter and temperatures may drop far below the melting point of their body fluids increasing the risk of internal ice formation. It is, therefore, of interest to understand how animals like enchytraeids can survive winters in these cold areas. Cold hardiness in enchytraeids has been reported in several studies, and they survive sub‐zero temperatures using either of the strategies *freeze tolerance* or *cryoprotective dehydration* (Kähler, 1970; Pedersen & Holmstrup, 2003; Slotsbo, Maraldo, Malmendal, Nielsen, & Holmstrup, 2008; Sømme & Birkemoe, 1997). Freeze tolerance can be defined as the process where the animal tolerates internal, but only extracellular, freezing of body fluids, which induces cellular dehydration (Zachariassen, 1985). In contrast, cryoprotective dehydration is a strategy where the entire animal becomes dehydrated when exposed to sub‐zero temperatures, which enables it to avoid freezing of body fluids (Holmstrup, Bayley, & Ramløv, 2002).

*Enchytraeus albidus* (Clitellata; Enchytraeidae) can be found in organic matter‐rich soils (e.g., in compost) as well as in decaying seaweed at the supralittoral zone along the coasts of Western Europe, Scandinavia, and Greenland (Fisker, Overgaard, Sørensen, Slotsbo & Holmstrup, 2014). This species is freeze tolerant and has a wide physiological tolerance range to freezing temperatures in its habitat (Slotsbo et al., 2008; Fisker et al. 2014). In general, freeze tolerance of ectothermic animals is promoted by adaptive modifications of membrane lipid composition that reduce the risk of deleterious phase transitions of cellular membranes during cold exposure, that is, homeoviscous adaptation (Kostal, 2010), and the accumulation of low molecular weight cryoprotectants, that can reduce the ice fraction at sub‐zero temperatures (Zachariassen, 1979). Clearly, a wide range of other molecular mechanisms is important for the ability to tolerate freezing of extracellular fluids in ectothermic organisms, but relatively little is known of this (Storey & Storey, 2013). Freeze‐tolerant animals promote freezing at high sub‐zero temperatures using ice nucleating proteins or inoculative freezing through the skin for controlled triggering of ice growth, and further may employ ice active (antifreeze) proteins that inhibit ice recrystallization. As cellular dehydration is an important consequence of freeze tolerance, aquaporins and other trans‐membrane channels that allow movement of cryoprotectants are important as well as cytoskeleton remodeling preserving cellular integrity. Further, anti‐oxidant defenses, heat shock proteins, and other chaperones are important for survival of freezing (for a review, see Storey & Storey, 2013).

In *E. albidus*, freeze tolerance involves active mobilization of glucose (from glycogen reserves) starting when the freezing process initiates and is therefore more akin to freeze tolerance of vertebrate ectotherms like frogs than of insects where cryoprotectant loading takes place months before the actual freezing of hemolymph starts (Fisker et al. 2014; Holmstrup, Costanzo, & Lee, 1999; Slotsbo et al., 2008). Strains of this species inhabiting different geographic locations have different freeze tolerance capacities. Arctic strains are—not surprisingly—clearly more freeze tolerant than strains of temperate regions and this is likely related to the capacity to adjust membrane phospholipid composition to low temperature, and the ability to accumulate large glycogen reserves during autumn. Glycogen is used for synthesis and accumulation of glucose serving as a cryoprotectant and as fuel during periods where the organism is frozen and unable to feed (Calderon, Holmstrup, Westh, & Overgaard, 2009; Fisker, Overgaard, Sørensen, Slotsbo & Holmstrup, 2014; Fisker et al., 2015; Slotsbo et al., 2008).

Studies at the transcriptomic level using *E. albidus* were first available in a lower throughput fashion in 2011, for example, the first in house microarray was developed by Amorim et al. (2011) to study the effects of chemical and natural stressors. This was further developed and used to explore the ecotoxicological mechanisms of several contaminants (Gomes, Soares, Scott‐Fordsmand, & Amorim, 2013; Novais, De Coen, & Amorim, 2012). Here, we attempt to obtain a more mechanistic understanding of freeze tolerance in *E. albidus* by studying differential transcriptomic responses between a freeze‐tolerant strain and a freeze‐sensitive strain. After de novo assembly and annotation of a consensus *E. albidus* transcriptome, two strains were compared using RNA sequencing; one originating from Greenland and one from Germany. The strain from Greenland tolerates much lower temperatures and endures freezing for much longer periods than worms from Germany (Fisker et al. 2014; Silva, Holmstrup, Kostal, & Amorim, 2013). Henceforth, we term the Greenland strain “freeze tolerant,” while the Germany strain is termed “freeze sensitive.” Our main aim was to identify key transcriptomic pathways linked to physiological alterations associated with freeze tolerance.

## 2 MATERIALS AND METHODS

### 2.1 Species

*Enchytraeus albidus* (Clitellata; Enchytraeidae) from Germany were obtained from a commercial supplier who originally collected the worms from garden compost (Büchner Zierfischfutter, Jena, Germany; coordinates: 51°51′N, 9°50′E). In our laboratory, the worms were cultured for 1 year in the laboratory in agricultural (loamy) soil at 5°C before being used in experiments. The worms were fed weekly with rolled oats mixed with dried and crushed macroalgae (predominantly *Fucus* spp., collected near Aarhus, Denmark). Worms from Greenland were collected in 2010 from decaying seaweed near the seashore at Kobbefjord about 20 km south‐east of Nuuk (coordinates: 64°8′N, 51°23′W). These worms were also kept in the laboratory as described for Germany worms for about 1 year prior to experiments. Two experts in enchytraeid taxonomy verified (based on morphology and anatomy characters) that both strains indeed were *E. albidus* (B. Christensen, personal communication; R. Schmelz, personal communication).

All experiments described in this study were performed using natural standard soil LUFA 2.2 (Speyer, Germany) This soil has ca. 6% clay, 17% silt, 77% sand, and 4.4% organic matter. The pH (CaCl2) of LUFA soil is 5.5, which is within the optimum pH range for *E. albidus* (Jänsch, Amorim, & Römbke, 2005).

### 2.2 Exposures

To ensure that we had broad representation of stress‐related genes in our reference transcriptome, we sequenced a pooled sample including mRNA from worms exposed to heat, cold, desiccation, pesticides, heavy metals, photoperiods, as well as worms at several developmental stages (adults, juveniles). Table S1 summarizes the exposures with relevant detailed exposure levels (Supporting Information). Worms were exposed following the standard guideline (OECD 2004) adapted to specific test treatment where needed. In short, ten adult worms with well‐developed clitellum were introduced into glass vessels (50 ml) containing 25 g of test soil (moistened to 50% of the water holding capacity). Four replicates per condition were used. The tests were run at 20 ± 1°C with a 16‐hr:8‐hr light:dark photoperiod (except when testing the different temperatures and photoperiod treatments, see Table S1).

### 2.3 Freezing experiment for RNA sequencing

Worms were either exposed to −5°C (hereafter termed “frozen”) or kept at 2°C as control (hereafter termed “control”)**.** Groups of ten adult enchytraeids were placed in 9‐ml vials containing approximately 5‐g fresh soil moistened with 0.22 ml tap water g^−1^ dry soil. The vials were closed with perforated lids (to allow ventilation) and kept at 2°C for 1 week prior to the freezing exposure. Freezing of the enchytraeids was accomplished by placing vials in a walk‐in freezer set to −1.5 ± 0.2°C. After 1 hr, a small ice crystal was added to the soil surface to ensure a controlled freezing of soil and worms at a high sub‐zero temperature (Slotsbo et al., 2008). After 16 hr, the soil and worms were frozen, and the vials were moved to a programmable cooling cabinet (Binder, Tütlingen, Germany) accurate to ± 0.2°C. The temperature was then gradually lowered to −5°C during 24 hr and kept at this temperature for 48 hr. With this freezing protocol, we simulated a harsh, but not unrealistic, freezing event in the natural habitat of Arctic *E. albidus* (Coulson et al., 1995). Frozen enchytraeids were sampled by quickly thawing the soil in cold tap water and blotting them dry with filter paper before they were placed in Eppendorf tubes. The tubes were snap frozen in liquid N_2_ and kept at −80°C until RNA extraction. Unfrozen control worms received the same treatment in identical vials, but were kept at 2°C, before snap freezing in liquid nitrogen for RNA extraction. Treatments and controls were replicated three times for freeze‐tolerant and freeze‐sensitive strains. It should be noted that both strains survive this freezing exposure (Fisker et al. 2014). Each replicate contained ten pooled animals to obtain sufficient RNA for sequencing.

### 2.4 RNA isolation, library prep, and sequencing

Total RNA was extracted using the TRIzol extraction method (Invitrogen, Belgium), followed by DNase treatment (Fermentas, Germany). The quantity and purity of the isolated RNA were measured spectrophotometrically on a NanoDrop ND‐1000 Spectrophotometer (Thermo Scientific), and its quality was checked on a denaturing formaldehyde agarose gel. In the case of Illumina Miseq sequencing, aiming to provide the basis for a complete transcriptome assembly, a pool was prepared including two replicates of each condition (depicted in Table S1), each replicate contributed 1 μg of Total RNA to the pool. Subsequently, Evrogen (Moscow, Russia) applied their SMART approach (Zhu, Machleder, Chenchik, Li, & Siebert, 2001), followed by their DSN normalization method (Zhulidov et al., 2004) to obtain a normalized cDNA library. The TruSeq mRNA library prep kit was used to prepare a normalized library for sequencing on a MiSeq. The Miseq version 3 kit was used according to manufacturer's instructions to sequence the normalized library for 300‐bp cycles paired end.

For the actual freezing experiment, aiming to obtain the freeze‐related transcriptomics, three replicate samples per treatment were selected and prepared for sequencing using the Illumina TruSeq^®^ RNA Library Preparation Kit v2 according to the protocol supplied by the manufacturer. Briefly, in this procedure, total RNA is purified for mRNA using oligo‐dT attached magnetic beads and mRNA is fragmented. Hereafter, mRNA is transformed into cDNA via reverse transcriptase and sample unique adapters are ligated to the cDNA fragments. Quality and quantity was assessed on a 2100 Bioanalyzer (Agilent). After an additional clean‐up procedure with magnetic beads, to remove traces of short adapter molecules, samples were diluted to 10 μmol/l cDNA and subsequently pooled for sequencing (100‐bp paired end). In total, 12 libraries were sequenced (three biological replicates of each treatment with each strain) on a single lane of an eight‐lane flow cell on the Illumina HiSeq 2500 platform at the VU Medical Center sequencing facility.

### 2.5 Assembly and data analysis

Sequence quality control on raw reads from both the MiSeq and the HiSeq runs was performed using FastQC. Raw read quality was high in general but the last 10 basepairs of the MiSeq reverse run were low in quality based on the calculated Phred score (inverse log of probability that the corresponding base call is incorrect). Consequently, low quality bases were trimmed using Trimmomatic with a quality cutoff set at 24 using Phred33 encoding according to Bolger, Lohse, & Usadel (2014). The transcriptome was assembled using Trinity RNA‐Seq (Haas et al., 2013) using the default k‐mer length settings. The two strains (Greenland and Germany) were assembled separately with each assembly containing the six strain‐specific samples combined with the MiSeq data. Each set of assembled contigs was further clustered using Cap3 software (Huang & Madan, 1999) in order to merge and extend the original contigs generated by Trinity. We compared contigs from each strain by running best reciprocal BLASTn software locally to define a core set of transcripts, which was used as mapping reference for each individual strain (Camacho et al., 2009). This core set of transcripts was tested for completeness and assembly redundancy using Benchmarking Universal Single‐Copy Orthologs (BUSCO) analysis against the metazoan database using default settings (Simão, Waterhouse, Ioannidis, Kriventseva, & Zdobnov, 2015). Subsequently, the core set was annotated using Blast2GO pro version 4.1 as follows. First, translated BLASTx was applied to identify homologs in the nonredundant NCBI sequence database, using expect‐value cutoff of 10^−5^ and at least 50% sequence similarity. Then, Gene ontology terms were mapped and annotated, including enzyme codes if available.

The mapping references for RNAseq expression analysis were generated using Bowtie2 (Langmead & Salzberg, 2012), while the actual mapping and read quantification was preformed with a combination of Bowtie2 and eXpress (Langmead & Salzberg, 2012; Roberts & Pachter, 2013). Statistical analysis of differential gene expression was performed in R using the EdgeR package (McCarthy, Chen, & Smyth, 2012), employing a GLM on a simple contrast in which the freezing treatment for each strain was directly compared to the control conditions. Moreover, the treatment x strain interaction was statistically tested. Subsequent *p*‐values were corrected for multiple testing using the False Discovery Rate (FDR) method (Benjamini & Hochberg, 1995). This yielded two sets of significant genes that were subjected to Gene Ontology (GO) analysis using the TopGO package in R (Alexa, Rahnenfuhrer, & Lengauer, 2006). For GO analysis, we focused on the Biological Process and Molecular Function components as these two are the most informative. GO terms which only contained one transcript were removed from the significant GO term list. Additional KEGG ortholog annotation was performed using the KEGG Automated Annotation Server (http://www.genome.jp/tools/kaas/), and gene expression pathway analysis was performed using the R‐packages GAGE in conjunction with Pathview using standard settings (Luo, Friedman, Shedden, Hankenson, & Woolf, 2009). We chose the four expression fold change ratios as determined by the gene expression interaction analysis from EdgeR as input for the KEGG pathway analysis in the GAGE package.

## 3 RESULTS AND DISCUSSION

The assembly and annotation of a reference transcriptome based on both MiSeq data and HiSeq data are presented first. The datasets were combined in order to generate a comprehensive transcriptome covering as many transcripts as possible. Subsequently, raw reads from the RNA sequencing experiment (HiSeq data) were mapped against the reference transcriptome to study differential gene expression. Finally, differential expression networks were associated with physiological alterations, using annotation information as well as pathway analyses.

### 3.1 Transcriptome assembly and annotation

For the assembly of the reference transcriptome, we made use of two Illumina datasets: The HiSeq run on the twelve barcoded treatment samples (i.e., two strains, frozen and control, three replicates) and a MiSeq run that contained a normalized pool of stress‐response samples (Table S1). Initially, we pooled all the data for a joined assembly, resulting in ~270 million quality‐controlled reads to be used in Trinity‐RNAseq, yielding in total 292,933 contigs with a median length of 413 basepairs. However, mapping of the two individual strain reads showed that the assembly was highly strain specific. As this strain‐specific assembly confounded gene expression interaction effects between strain and treatment, we decided to assemble the strains separately and use best reciprocal BLAST‐hit (Moreno‐Hagelsieb & Latimer, 2007) to define a core transcriptome. In this core transcriptome, genes have a specific sequence for each strain but are linked between strains as orthologs. The strain‐specific assembly yielded 56,050 contigs (median length of 846 bp) for the Germany strain and 51,918 contigs (median length of 845 bp) for the Greenland strain. Subsequent best reciprocal BLASTn analysis between the two strains yielded a final core transcriptome set of 40,002 genes with an average median length of 950 bp, which was used as mapping reference for the differential gene expression analysis (Table 1).

**Table 1:** Summarized properties of assembly, annotation, and quality control of the core transcriptome used for mapping of the RNA sequencing data for Germany strain (G) and Greenland strain (GR)

	G‐strain	GR‐strain
Total sequences	40,002	40,002
Total bases (Mbp)	52.41	49.28
Min length (bp)	201	203
Max length (bp)	24,453	23,568
N50 length (bp)	1,744	1,664
Mean length (bp)	1,310	1,232
GC%	41.07	41.05
BLAST‐hit	13,231 (34%)	12,799 (32%)
Gene Ontology	12,994 (32%)	12,468 (31%)
KEGG pathway	9,964 (25%)	9,922 (25%)
BUSCO complete	84.10%	82.40%
BUSCO complete and also single copy	75.40%	76.10%

The two gene sets were annotated with a combination of BLASTx and Blast2GO and yielded 13,231 genes with a blast result (34%) for the Germany strain and 12,699 genes with a blast result (32%) for the Greenland strain (Table 1). About 32% of the genes which contained a BLAST‐hit in the Germany strain were fully annotated and usable for Gene Ontology (GO) analysis. The transcriptome of the Greenland strain contained 31% of fully annotated genes, and KEGG pathway information could be retrieved for 25% of all assembled contigs in both strains (Table 1). As the two sets were considered ortholog sets with highly similar annotation information, we merged the annotation results for GO analysis and pathway analysis to obtain an unbiased set of annotation terms per gene to be used for both strains. The combined annotation also yielded a total of 1,136 unique enzyme codes which could be mapped to metabolic pathways using IPATH to determine the completeness of metabolic pathways represented in the transcriptome (Letunic, Yamada, Kanehisa, & Bork, 2008). This is visualized as red edges in Fig. S1, suggesting that most of the metabolic pathways are represented except for metabolism of terpenoids and polyketides, which are usually more associated to microbes and plants. The carbohydrate and fatty acid metabolic pathways, which are involved in mechanisms of freeze tolerance, are well represented. Finally, transcriptome completeness was determined by applying BUSCO analysis, using a curated reference set of single‐copy core genes present in all metazoans. This assessment shows that about 84% of this core set is identified in the current *E. albidus* transcriptome, indicating a high level of completeness (Table 1). We have to note, however, that about 9% of these core set genes are present as duplicates. This suggests that the assembly process generated some redundancy within the final set of contigs (Simão et al., 2015).

### 3.2 Differential gene expression analysis

**Figure 1:**
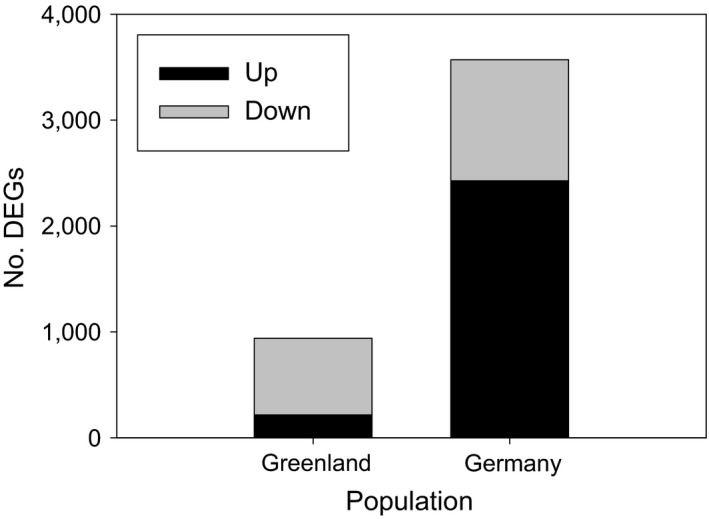
Total number of differentially expressed genes (DEGs) in response to freeze treatment (−5°C) as compared to control/unfrozen (+2°C) temperature at false discovery rate level of *p* < .05, including the respective numbers of up‐ and down‐regulated transcripts; In the case of Greenland 215 transcripts are up‐regulated, and 725 transcripts are down‐regulated, while for Germany population, 2,426 transcripts are up‐regulated, and 1,143 transcripts are down‐regulated

**Figure 2:**
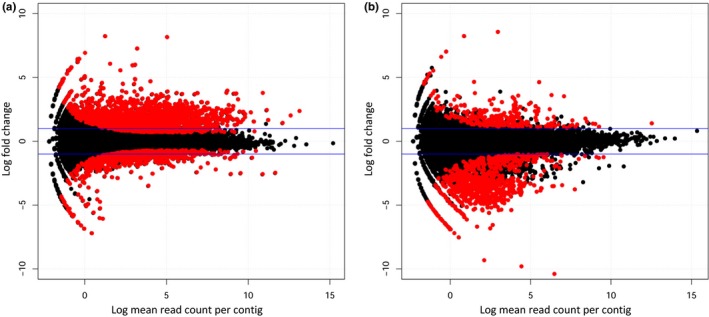
Smear plots of differential gene expression of freeze treatment (−5°C) versus control (+2°C). (a) Germany freeze‐sensitive strain; (b) Greenland, freeze‐tolerant strain. *X*‐axis, Log mean read count per contig (CPM). *Y*‐axis, Log fold change (FC). Red dots, significantly differentially expressed contigs (FDR corrected *p* < .05); black dots, nonsignificantly differentially expressed contigs

Each RNAseq library generated on average 20.3 million of paired‐end raw sequence reads per sample, of which 97% passed quality control. After mapping of the RNAseq reads of the Germany strain to the assembled contigs, we identified 3,569 transcripts to be differentially expressed between unfrozen/control conditions (+2°C) and the frozen treatment (−5°C). From these, 2,426 transcripts were significantly up‐regulated, while 1,143 were significantly down‐regulated (Figure 1). In case of the Greenland strain, we identified only 940 transcripts to be significantly differentially expressed between unfrozen (controls) and frozen worms. Only 215 transcripts were significantly up‐regulated, while 725 were significantly down‐regulated (Figure 1). Figure 2 shows the smear plots, visually illustrating the differences between the two strains, which already indicates that the transcriptome profile of the freeze‐tolerant strain represents a phenotype that is much less perturbed due to freezing, when compared to the freeze‐sensitive Germany strain. This lack of perturbed transcriptional profile has been observed regularly both in animals and plants (Roelofs, Aarts, Schat, & Van Straalen, 2008), and thus seems to represent a general pattern of tolerance. It reflects the ability of a tolerant phenotype to maintain homeostasis under circumstances that would be considered as stressful for nonadapted organisms. Several examples show that this is a consequence of the alteration to increased constitutive expression of otherwise stress‐inducible genes coding for enzymes/peptides that protect the organism against environmental stress (Halimaa et al., 2014; Roelofs et al., 2009).

Functional annotation and GO term enrichment analysis of significantly different transcripts show that the up‐regulated genes in the Germany strain can be linked to changes in lipid metabolism, while significantly down‐regulated genes are mainly related to mRNA processing and splicing (Table S2). In contrast, significantly up‐regulated genes in the Greenland strain are mainly associated with transport of sodium and drugs as well as more general processes related to transcriptional regulation. In several arthropods, sodium/calcium transporters are up‐regulated in response to cold (Des Marteaux, Mckinnon, Udaka, Toxopeus, & Sinclair, 2017; Gao et al., 2009). In situ hybridization showed that increased sodium transport was localized at the nephridial canal, a region previously associated with ion reabsorption (Gao et al., 2009). Cold acclimation in trout is also associated with increased sodium pump activity in erythrocytes. The authors suggested that this compensates for the reduced trans‐membrane gradients of sodium and potassium in erythrocytes during cold temperature regimes (Raynard & Cossins, 1991). In insects, however, cold exposure can result in chill coma and chilling injury, which is related to reduced active ion transport. This causes leaking of cellular K^+^ and depolarization of the resting potential, which ultimately can result in influx of Ca^++^, apoptosis and necrosis (Overgaard & MacMillan, 2017). In freeze‐tolerant *E. albidus*, these physiological constraints are also a threat for survival, but there might be additional problems with pumping ions in a frozen state when cells are dehydrated and much of the extracellular fluids are changed to ice. We cannot say whether the differential expression of genes coding for the mentioned ion transporters we observed is directly related to cold injuries, but the results do suggest that differences in freeze tolerance, in addition to many other processes, may also somehow be associated with the expression of ion pumps. More targeted studies are needed to look into this.

### 3.3 Population × freeze treatment interaction

**Figure 3:**
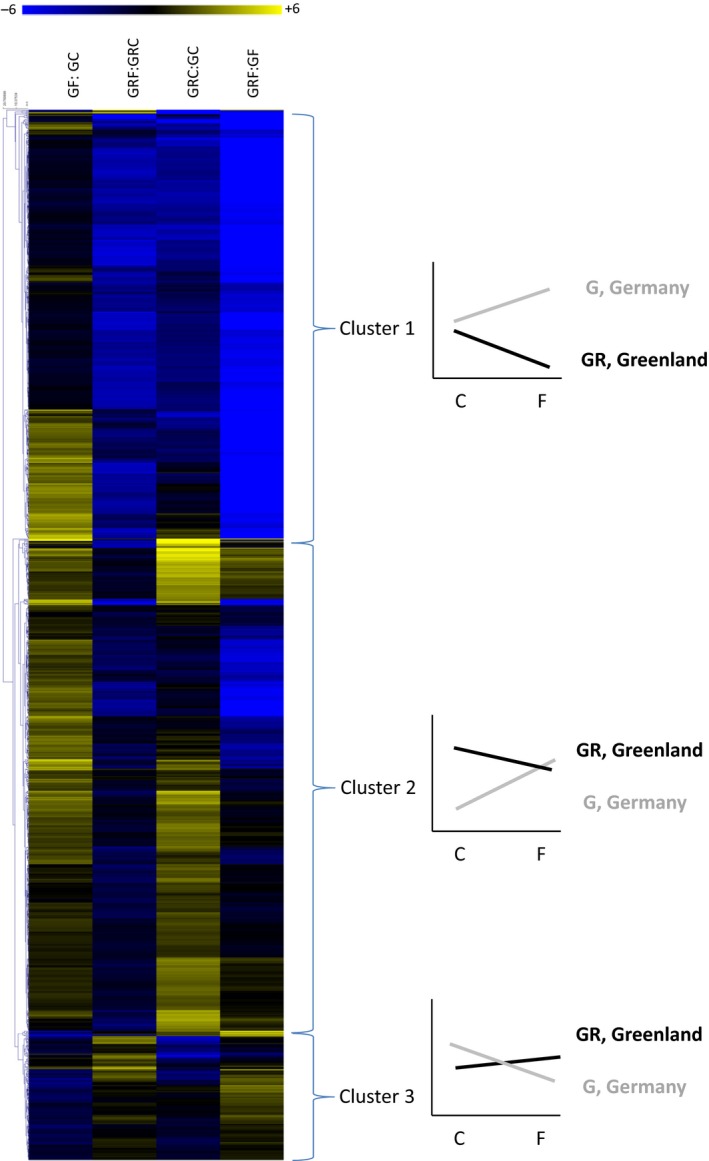
Heat plot of all significant gene transcripts. Blue color, down‐regulation (log2 fold change), yellow color, up‐regulation (log2 fold change). C, control/unfrozen at +2°C, F, Frozen at −5°C. GC, Germany control; GF, Germany frozen; GRC, Greenland control; GRF, Greenland frozen. The three clusters depict general patterns of gene regulation with its representative gene regulation pattern next to each cluster. Black lines, regulation of Greenland strain; gray lines, regulation of Germany strain

By comparing the differential expression profiles of the freeze‐tolerant Greenland strain with the freeze‐sensitive Germany strain, we were able to identify genes and molecular processes that changed during freezing and were different in the two strains. These gene sets may have undergone transcriptional regulatory evolution in order to genetically adapt to more frequent and more severe freezing conditions in the Greenland strain. To that end, we statistically analyzed transcripts that show an interaction between genotype (Greenland versus Germany) and response to freezing (control versus frozen). In total, 1,374 transcripts showed such significant interaction (Table S3). Figure 3 shows the heat plot after clustering of these transcripts. In general, three main clusters can be distinguished. Cluster 1 consists of 560 transcripts that are slightly up‐regulated or nonresponsive to freezing in the Germany strain, but show clear down‐regulation in the Greenland strain. Moreover, all transcription levels are higher in the Germany strain when compared to the Greenland strain. Cluster 2 comprises 645 transcripts that are highly inducible in the Germany strain, but are slightly down‐regulated or nonresponsive in the Greenland strain. Finally, cluster 3 is the smallest group containing 169 transcripts that are down‐regulated in the Germany strain, but slightly up‐regulated in the Greenland strain, while total transcript level of frozen worms is higher in the Greenland strain than in the Germany strain.

Gene ontology enrichment analysis was performed on the annotated transcripts belonging to each of the three clusters. Transcripts of cluster 1 could be grouped into blood coagulation, chromatin condensation, and positive regulation of transcription. Transcripts belonging to cluster 2 were linked to lipid metabolism (long‐chain fatty acid biosynthesis), oxidation reduction, and metabolism and transport of anions, lipids, and drugs. Unfortunately, the level of annotation in cluster 3 was too low to provide significantly enriched processes. However, a member of the HSP40/DnaJ family (DnaJB2 homolog) was identified, which is down‐regulated in the Germany strain, slightly up‐regulated in the Greenland strain, but shows a high constitutive expression level in the Greenland strain (transcript EAID 31697, Table S3). This family of molecular chaperones engages in a complex with Heat shock protein 70, thereby stimulating its adenosin triphosphate domain (ATPase) providing substrate specificity of Hsp70 (Ohtsuka & Hata, 2000). Interestingly, we identified a transcript in cluster 2, homologous to hsp70 (transcript EAID_14676, Table S3). This transcript was up‐regulated in the Germany strain and nonresponsive to freezing in Greenland strain, but constitutively overexpressed when compared to Germany. This implies that heat shock proteins such as Hsp70 are relevant in adaptation to cold environments and the risks associated with freezing of body fluids. This interpretation is in accordance with studies of other freeze‐tolerant invertebrates. Rinehart et al. (2006) have shown that larvae of the freeze‐tolerant Antarctic midge, *Belgica Antarctica*, have a continuous up‐regulation of hsp70 even during the summer period when there is no risk of freezing.

### 3.4 Linking differential expression to physiological alterations

When comparing gene expression of frozen and unfrozen control animals recall that the “frozen” treatment will potentially include mRNA transcribed both during the freezing process itself (at −1.5°C) and/or during the subsequent 72 hr where animals were kept frozen at −5°C. The mRNA abundance therefore represents a response to the freezing process where ice formation and cell shrinking takes place, but apparently inducing transcriptional changes even though cells were dehydrated. Previous studies showed that ice fraction at −5°C is relatively low due to accumulation of high glucose concentrations allowing for a significant metabolic rate at this temperature, and therefore probably also allowing for a significant rate of differential transcription (Fisker et al. 2014; Patrício Silva, Enggrob, Slotsbo, Amorim, & Holmstrup, 2014). Still, we are careful with the interpretation of the observed differential transcription. For example, we can only assume that this mRNA has been translated into protein. Alternatively, the mRNA was regulated in anticipation of the processes associated with thawing, but our study does not allow for teasing apart these two different possibilities.

Freeze tolerance is accomplished by a complex physiological process involving several pathways acting in concert. Our transcriptomic study underpins several physiological responses to cold exposure and freezing that have been observed in *E. albidus*. By linking the molecular transcriptome level to the physiological/cellular level, we provide an additional verification step toward the identification of pathways that have undergone adaptive evolution. Several processes seem to have been evolving by natural selection driven by freezing conditions in the habitats of this species. Fatty acid, glucose, glycogen metabolism, and oxidative stress defense mechanisms were previously suggested to be involved in freeze tolerance in this species. We will show results and discuss these processes in the context of molecular pathways identified by the interaction analysis between treatment and strain within the RNAseq data (Figure 3).

### 3.5 Fatty acid metabolism

**Figure 4:**
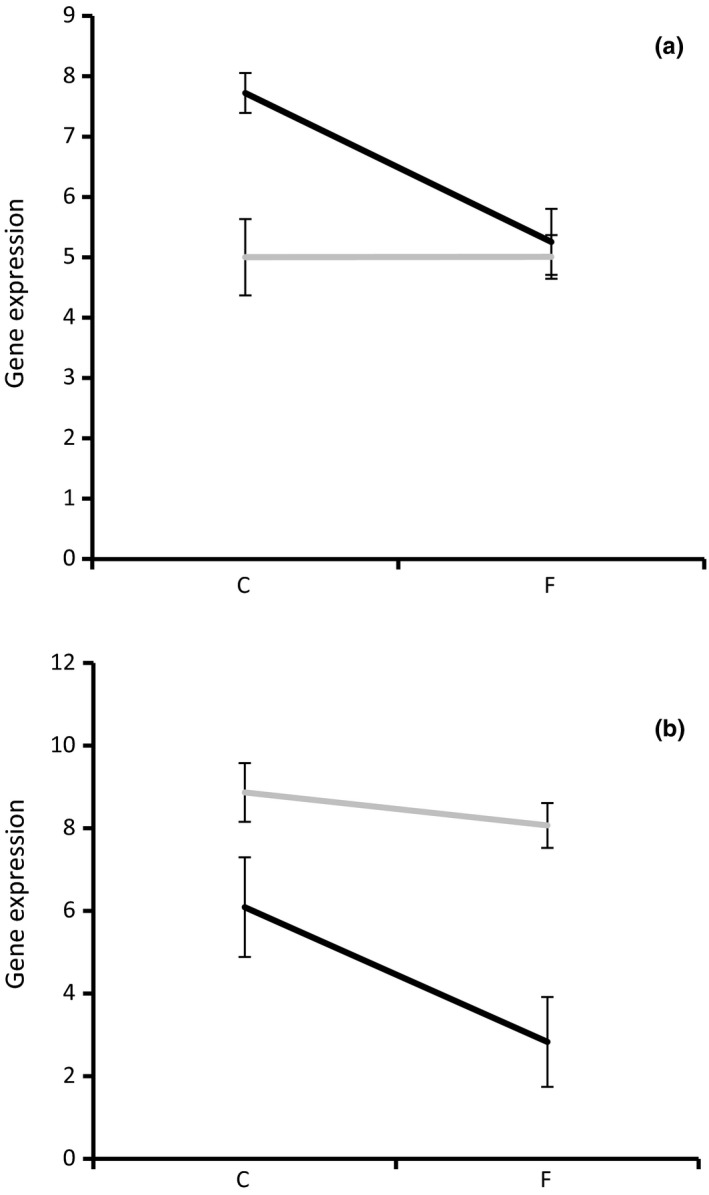
Representative gene expression patterns for interaction cluster 2 (see Figure 3). (a) Very long‐chain fatty acid elongase protein 3; (b) Glycogenin‐1. Black line Greenland strain; gray line, Germany strain. *Y*‐axis, Log2 normalized gene expression measured as effective RNAseq read counts; *x*‐axis temperature treatments (C, control/unfrozen +2°C, F frozen treatment −5°C). Error bars represent standard deviations in gene expression

The GO enrichment analysis already indicated that fatty acid metabolism was significantly affected (Table S2), suggesting that gene expression alterations underpin the importance of this process in adaptation to cold. Eight genes were identified within Cluster 2 for which the gene expression patterns show a significant interaction between freeze treatment and strain (Table 2). They code for fatty acid synthase, two very long‐chain fatty acid elongases, fatty acid desaturase, Omega 6 fatty acid desaturase, fatty acid lipase, and fatty acid acyl reductase. Figure 4a shows the expression pattern of very long‐chain fatty acid elongase protein 4, which is a representative pattern for all eight genes. Overall, they are down‐regulated in the freeze‐tolerant Greenland strain when compared to the freeze‐sensitive Germany strain, although the absolute value of the transcriptional level is higher in the Greenland strain (Figure 4a).

**Table 2:** Differential expression values (Log 2 ratio) for genes involved in fatty acid metabolism. GF, Germany frozen (−5°C), GC Germany control/unfrozen (+2°C). GRF, Greenland frozen (−5°C); GRC Greenland control/unfrozen (+2°C). FAS, fatty acid synthase. FA ligase, fatty acid ligase

	GF:GC	GRF:GRC	GRC:GC	GRF:GF
FAS	−0.177	−4.286	6.242	2.133
Desaturase	−0.313	−2.588	3.966	1.69
Elov4	0.514	−1.506	3.763	1.744
Omega 6 desaturase	−0.36	−2.83	3.084	0.614
Acyl reductase	0.266	−2.094	3.014	0.653
Elongase 3	0.029	−2.499	2.869	0.34
FA ligase	1.448	−1.134	2.607	0.026
Elov6	0.3	−1.804	2.399	0.296

Genes involved in fatty acid metabolism were negatively affected at the transcriptional level in the Greenland strain. However, they maintain a higher level of transcriptional activation in all cases, when compared to the levels in the sensitive strain. This suggests that membrane lipid modifications are ongoing even in the frozen state at −5°C, and at a higher level than in the Germany strain. Fisker et al. (2015) analyzed the composition of membrane phospholipid fatty acids (PLFAs) in several geographically different strains of *E. albidus*. They showed that the preponderance of shorter unsaturated PLFAs (e.g., linoleic acid) is associated with increased freeze tolerance of strains from arctic regions, a pattern that has also been observed in *Drosphila* species (Slotsbo et al., 2016). Here, we provide evidence that several fatty acid elongases are being suppressed in the freeze‐tolerant Greenland strain upon freezing treatment, while these genes remain unchanged in the German strain. This suggests that the freeze‐tolerant Greenland strain potentially is more flexible in controlling PFLA length over a wide temperature gradient. However, as we have no evidence that transcription of these genes actually results in protein synthesis responsible for PLFA modifications in the frozen state, our results cannot be directly used as molecular support to observations showing that freeze‐tolerant strains of *E. albidus* have higher proportions of short chain PLFAs than freeze‐sensitive strains (Fisker et al., 2015).

### 3.6 (Poly)sugar metabolism and transport

We annotated four genes involved in glucose metabolism and transport in cluster 2 (Figure 3), among which are two sodium‐dependent glucose transporters, glucose dehydrogenase, and glucose isomerase (involved in glycolysis). Their expression patterns are highly comparable to the expression patterns observed among genes involved in fatty acid metabolism. During freeze treatment these genes are slightly down‐regulated in the Greenland strain, but the absolute levels of gene expression are mostly significantly higher when compared to the Germany strain.

In contrast, glycogenin‐1, a protein involved in glycogen biosynthesis, is less expressed in the Greenland strain when compared to the Germany strain. Upon freezing treatment, this gene is strongly down‐regulated in the Greenland strain, while expression levels in the Germany strain remain high.

Glucose is a well‐known cryoprotectant in oligochaetes, because it is the primary blood sugar of these animals (Holmstrup et al., 1999). Glucose transport seems to be decreased, and glucose break down (glucose dehydrogenase activity and glucose isomerase activity) seems to be decreased as well. This is in line with previous observations from Slotsbo et al. (2008), that glucose levels are accumulating in the Greenland strain at the expense of glycogen levels to serve as a cryoprotectant. This conclusion is further underpinned by a severe down‐regulation of glycogenin‐1 in the tolerant strain (Figure 4b). This protein is essential in glycogen biosynthesis, indicating that glycogen biosynthesis is down‐regulated to keep glucose levels high for optimal cryoprotection. It also shows again that Greenland worms respond much more flexible to temperature changes when compared to the Germany strain with regard to physiological processes (PFLA composition, glucose content) essential in coping with below zero temperatures. The level of plasticity in these responses may be an important genetic adaptation to withstand fluctuating freezing temperatures, which maybe a realistic scenario under natural conditions.

### 3.7 Oxidative stress

**Figure 5:**
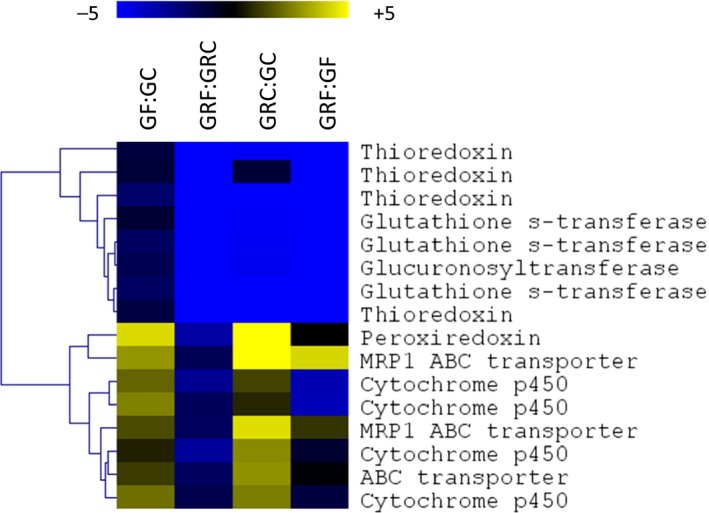
Heat plot of differential gene expression (Log2 ratio) of genes involved in oxidative stress. Blue: down‐regulation; Yellow: up‐regulation. GF; Germany frozen at −5°C, GC: Germany strain control/unfrozen condition +2°C. GRF: Greenland frozen at −5°C; GRC: Greenland control/unfrozen at +2°C

Genes involved in oxidative stress were observed both within cluster 1 and cluster 2. Cluster 1 (Figure 3) contained gluthatione‐S‐transferases, thioredoxins, and glucuronosyltransferase. Overall, gene expression levels were always significantly lower in the Greenland strain when compared to the Germany strain, and the freezing treatment caused an increase in this differential expression pattern. Cluster 2 contained several ABC transporters and Cyp p450s (Table S3, Figure 5). These genes were all slightly down‐regulated due to freezing in the Greenland strain, while being slightly up‐regulated in the Germany strain. They suggest that the Greenland strain encounters less oxidative stress than the Germany strain, although ABC transporters and Cyp p450s may not necessarily always be associated with oxidative damage. Nevertheless, this effect becomes even more pronounced under freezing conditions.

Response to oxidative stress is a very general process associated with almost all types of stress (Tomanek, 2015). Silva, Holmstrup, and Amorim (2013) showed a higher activity of superoxide dismutase as well as high levels of glutathione in freeze‐tolerant Greenland worms, when compared to Germany strain. This indicates a higher capacity of tolerant phenotypes to withstand oxidative stress during freezing. Here, we observed genes coding for thioredoxins and glutathione‐S‐transferases to be down‐regulated in the Greenland strain, when compared to the Germany strain (Figure 5). This may support the observation of Silva, Holmstrup, & Amorim, 2013 that these worms suffer much less from oxidative stress when frozen, but we did not investigate the abundance and activity of the enzymes and the actual balance of ROS production. Moreover, the amplitude of transcriptional variance in response to freezing is much higher in freeze‐tolerant phenotypes when compared to freeze‐sensitive animals (Figure 5), suggesting that plasticity of transcriptional regulation of freeze tolerance‐associated gene networks is an adaptive feature of freeze‐tolerant phenotypes.

Interestingly, two metallothionein (mt) transcripts were significantly up‐regulated only in the freeze‐tolerant Greenland strain. Metallothionein is mainly involved in metal detoxification, but can also be activated by oxidative stress (Haq, Mahoney, & Koropatnick, 2003; Ling et al., 2016). This was recently observed in the earthworm, *Dendrobaena octaedra*, in response to freezing (Fisker, Holmstrup, & Sorensen, 2016), and the authors proposed two scenarios to explain this result. Metal ion concentrations are expected to increase rapidly upon freeze‐induced dehydration of the cell, which could trigger mt activation. Alternatively, mt could be induced by hypoxia as a result of reduced diffusion of oxygen into the worm body. Anoxic conditions lead to increased ROS, while two previous studies showed that mt can act as anti‐oxidant agent (English & Storey, 2003; Viarengo et al., 1999). Thus, mt induction in the Greenland strain may be an indirect response to oxidative stress and/or increased metal ion concentrations at the cellular level.

### 3.8 Pathway analysis

**Figure 6:**
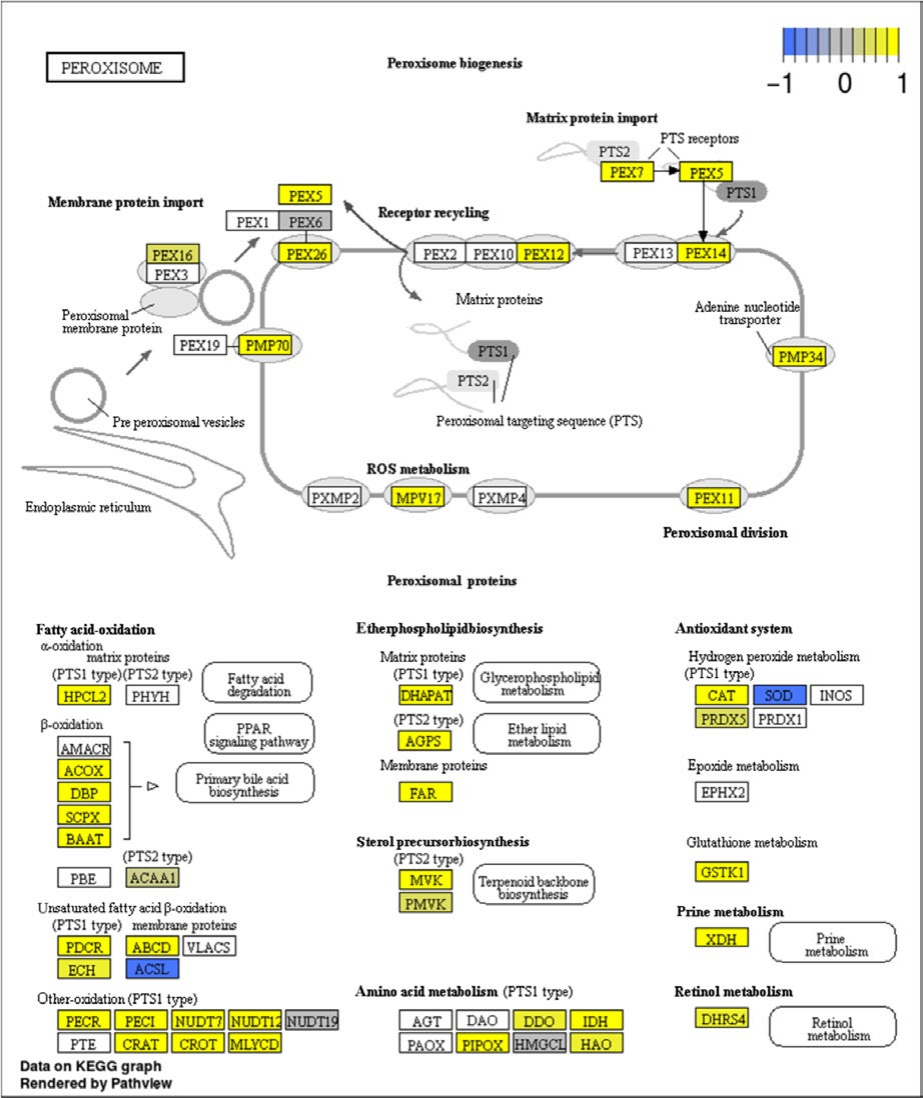
Differential expression of genes representing components of the “Peroxisome” KEGG pathway, as an example of pathways that are significantly differentially expressed between freeze‐tolerant Greenland strain and freeze‐sensitive German strain, in this case under frozen conditions (−5°C). Yellow color indicates up‐regulation in Greenland strain; Blue color indicates down‐regulation of Greenland strain. PEX, peroxisine; PMP, ABC transporter subfamily D; MPV, glomerulosclerosis gene Mpv17; HPCL, 2‐hydroxyacyl‐CoA lyase 1; DHAPAT, glyceronephosphate O‐acyltransferase; ACOX, acyl‐CoA oxidase; DBP, 3‐hydroxyacyl‐CoA dehydrogenase; SCPX, sterol carrier protein 2; BAAT, bile acid‐CoA amino acid N‐acyltransferase; ACAA1, acetyl‐CoA acyltransferase 1; AGPS, alkyldihydroxyacetonephosphate synthase; FAR, alcohol‐forming fatty acyl‐CoA reductase; MVK, mevalonate kinase; PMVK, phosphomevalonate kinase; CAT, catalase; PRDX, peroxiredoxin; SOD, superoxide dismutase; GSTK1, glutathione‐S‐transferase kappa 1; PDCR, peroxisomal 2,4‐dienoyl‐CoA reductase; ABCD, ABC transporter; ECH, Delta(3,5)‐Delta(2,4)‐dienoyl‐CoA isomerase; ACSL, long‐chain acyl‐CoA synthetase; XDH, xanthine dehydrogenase/oxidase; PECR, peroxisomal trans‐2‐enoyl‐CoA reductase; PECI, peroxisomal 3,2‐trans‐enoyl‐CoA isomerase; NUDT, peroxisomal coenzyme A diphosphatase; CRAT, carnitine O‐acetyltransferase; CROT, carnitine O‐octanoyltransferase; MLYCD, malonyl‐CoA decarboxylase; DDO, D‐aspartate oxidase; IDH, isocitrate dehydrogenase; PIPOX, L‐pipecolate oxidase; HAO, (S)‐2‐hydroxy‐acid oxidase; DHRS4, dehydrogenase/reductase SDR family member 4. Details in this pathway can be retrieved from the following website: http://www.genome.jp/kegg-bin/show_pathway?ko04146

In total, 56 pathways showed a significant interaction between population and freeze treatment (Table S4). The majority of these (51) are represented by higher expression in freeze‐tolerant Greenland strain as compared to Germany. When considering pathway functions, we observe 21 of the 56 pathways to be involved in cell signaling. This is in accordance with the study on cold acclimation in fall field cricket, where most of the up‐regulated pathways were represented by signaling as well (Des Marteaux et al., 2017). Also, in line with this study was the discovery of the endocytosis pathway, up‐regulated in the adapted Greenland strain. Remarkably, five significant pathways are associated with lipid metabolism, and up‐regulated in the Greenland strain when compared to the German strain under control conditions. This supports earlier descriptions in this study, based on differential expression of individual genes (Table 2, Figure 4), and underpins the relevance of those pathways in freeze tolerance. The majority of enzymes acting in the peroxisome are also up‐regulated in the Greenland strain both under control and freezing conditions (Figure 6). Peroxisomes are important organelles providing different metabolic activities, such as lipid oxidation, reduction in reactive oxygen species, and biosynthesis of phospholipids. Finally, we note that oxidative phosphorylation and Toll‐like receptor pathway are significantly up‐regulated in Greenland under freezing conditions. Several previous studies suggest that insects increase immunity after cold exposure (Sinclair, Ferguson, Salehipour‐shirazi, & MacMillan, 2013). Our data suggest that innate immunity may play an important role in freeze tolerance in *E. albidus*. Whether this is a nonadaptive by‐products of a generalized stress response, or the result of a coadaptation linking pathogens and cold remains to be elucidated in the future. In summary, the pathway analysis suggests that the freeze‐tolerant strain is able to maintain important metabolic functions, such as energy (ATP) production as well as innate immune responses even under freezing conditions.

## 4 FUTURE DIRECTIONS

Our study is one of the first employing a global transcriptomics approach to the elucidation of freeze tolerance physiology in invertebrates. We identified several major responses to freezing that deserve further investigation in more targeted studies. Recent studies suggest that tolerance of freeze‐thaw events in enchytraeids has a significant genetic component (Fisker et al. 2014). The present study suggests these genetic differences have a basis in the expression of molecular chaperones, and pathways involved in fatty acid metabolism, cell signaling, energy metabolism, innate immunity, and reduction of oxidative stress caused by freezing and possibly thawing. Such issues would be important when assessing the potential threats of climate change driving increased frequency of freezing and thawing events which may threaten soil biota in the Arctic and subarctic areas.

## 5 CONCLUSION

Overall, we observed two patterns of gene expression regulation in freeze‐tolerant animals. One pattern resembles constitutive overexpression of otherwise highly inducible stress response genes like Hsp40/DnaJ and Hsp70. Such patterns have been described in several previous studies dealing with genetic adaptation to stress tolerance. Secondly, the freeze‐tolerant phenotype shows plastic responses in transcriptional regulation of genes involved in pathways that were earlier described to tolerate freezing conditions in physiological studies (PFLA composition, sugar metabolism, and oxidative stress). This flexibility is proposed to maintain homeostasis under freezing conditions, which is observed at the physiological level as well as the transcriptome level. This apparently results in much less overall transcriptional perturbation in the freeze‐tolerant phenotype, while strong perturbation of the complete transcriptional machinery is observed in reference to *E. albidus* animals under realistic freezing conditions. We speculate that this may be the consequence of the type of stressor. Temperature fluctuations are of a seasonal and diurnal nature, requiring highly plastic phenotypic response. As such, increased plasticity in protective cascades may be much more effective to counteract the physiological challenges incurred by freezing.

## CONFLICT OF INTEREST

None declared.

## AUTHOR CONTRIBUTIONS

MJBA, MH, DR, and TEdB concepted and designed the work. MJBA, MH, DR, RV, and TEdB collaboratively drafted the manuscript and critically revised subsequent manuscript versions for important intellectual content. RV and MJBA isolated RNA, and RV prepared Illumina Truseq sequence libraries. TEdB and DR analyzed RNAseq data and performed statistical analysis as well as pathway analysis. MH performed field sampling. MH and MJBA performed stress and freeze treatments. All authors are accountable for all aspects of the work and ensure that questions related to the accuracy or integrity of any part of the work are appropriately investigated and resolved.

## DATA ACCESSIBILITY

All raw RNAseq data as well as all assemblies are submitted to NCBI under BioProject ID code PRJNA386732.
