# Supplementary figures and images for: Population‐specific transcriptional differences associated with freeze tolerance in a terrestrial worm
Source: Ecol Evol. 2018 Mar 11;8(7):3774–86. doi: 10.1002/ece3.3602 (PMC5901168; doi:10.1002/ece3.3602)

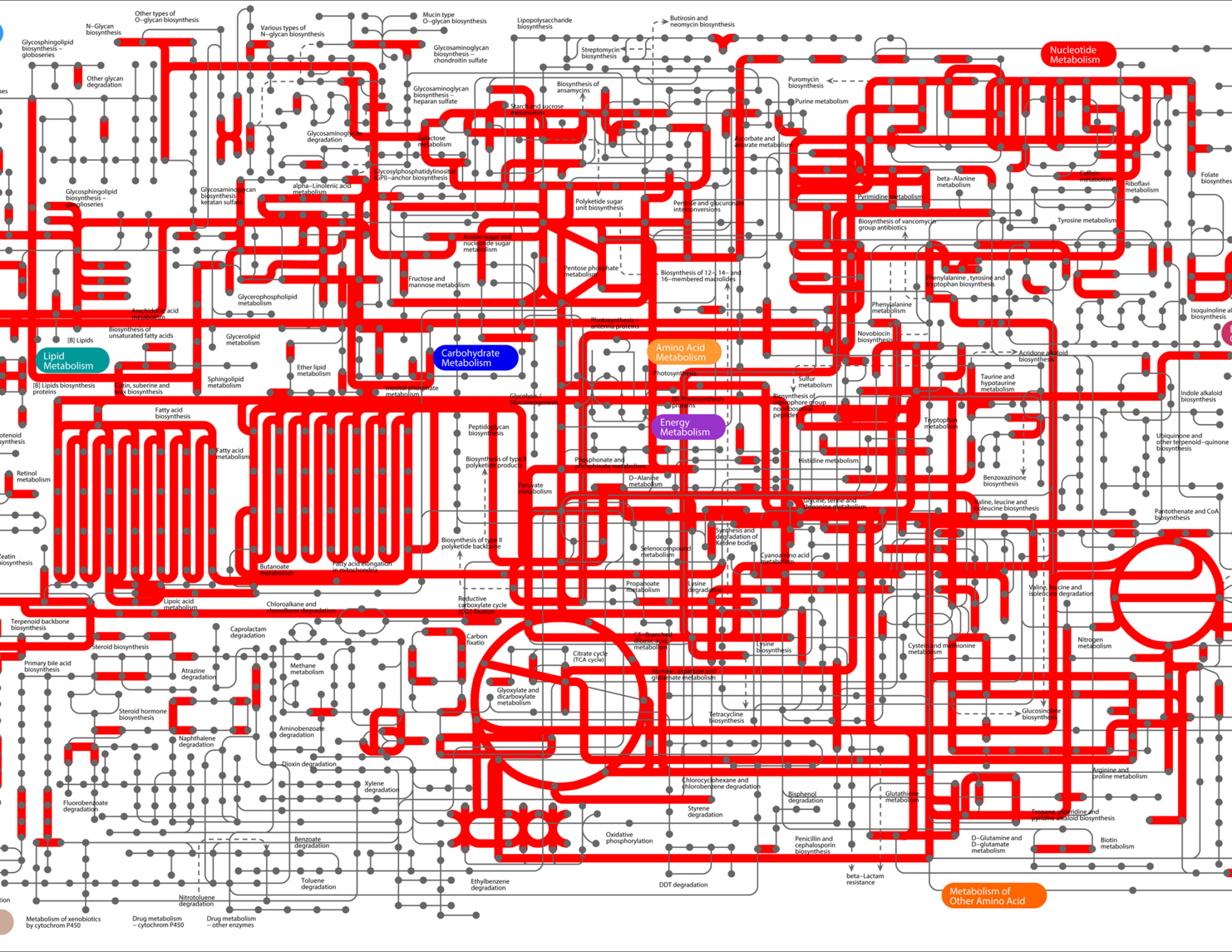

Supplement: Supplementary file 1 [file ECE3-8-3774-s001.pdf]
