# Supplementary material for: Population‐specific transcriptional differences associated with freeze tolerance in a terrestrial worm
Source: Ecol Evol. 2018 Mar 11;8(7):3774–86. doi: 10.1002/ece3.3602 (PMC5901168; doi:10.1002/ece3.3602)
Supplement: Supplementary file 2 [file ECE3-8-3774-s002.docx]

**Table S1**: Summary of the exposure conditions performed using 2 populations of *Enchytraeus albidus*, N: Nuuk (Greenland) and G (Germany). Information about the test treatments is given including exposure temperature (ºC), salinity (‰) time (days), desiccation (% relative humidity), photoperiod (light:dark), developmental stages (adults, juveniles), chemicals and concentrations. (grey shaded temperature were the further studied treatments).

| **Test treatment** |  |  |  |
| --- | --- | --- | --- |
| **Temperature (ºC)** | **Days** | **concentration** | **Population** |
| 5 (from cultures) | 0 | - | N+G |
| 30 (2 hrs) | 0,08 | - | G |
| 20 (from cultures) | 2 | - | G |
| 2 | 14 | - | G |
| -5 | 3 | - | N+G |
| **Salinity (‰)** (at 5ºC) | 2 | 35‰ | N+G |
| **Desiccation (**3 days at 5°C) | 2 | 98% RH | G |
| **Photoperiod** (at 20ºC) | 2 | Total dark | G |
|  | 2 | Total light | G |
| **Developmental stages** |  |  |  |
| Adults (from cultures) | 2 | - | G |
| Juveniles (from cultures) | 2 | - | G |
| **Chemicals** |  | **concentration** (mg/kg) |  |
| CuCl_2_.2H_2_O | 4 | 100 | G |
| CdCl_2_ | 2 | 6 | G |
| AgNO_3_ | 2 | 25 | G |
| NiCl_2_.6H_2_O | 2 | 50 | G |
| ZnCl_2_ | 4 | 40 | G |
| Atrazine | 2 | 3 | G |
| Dimethoate | 2 | 2 | G |
| Carbendazim | 2 | 0.5 | G |
| 4-Nonylphenol | 2 | 100 | G |
| Boric acid | 2 | 1000 | G |
| Acetone | 2 | 100% (same volume as added as chemical carrier) |  |
